# On the $L^2$-norm of Gegenbauer polynomials

**DOI:** 10.1007/s40096-021-00398-1

**Published:** 2021-04-21

**Authors:** Damir Ferizović

**Affiliations:** Graz University of Technology Institute of Analysis and Number Theory, Kopernikusgasse 24/II, 8010 Graz, Austria

**Keywords:** Gegenbauer polynomials, $L^2$-norm, Asymptotics, 33C45, 33F99, 41A60

## Abstract

Gegenbauer, also known as ultra-spherical, polynomials appear often in numerical analysis or interpolation. In the present text we find a recursive formula for and compute the asymptotic behavior of their $L^2$-norm.

## Introduction

Gegenbauer polynomials $\mathcal {C}_{n}^{(\lambda )}$, where $\lambda \in I_G:=\,(-\frac{1}{2},0)\cup (0,\infty )$ is called the index and $n\in \mathbb {N}_0$ is the degree, are the coefficients of following power series expansion in $\alpha$:

$$\begin{aligned} (1-2x\alpha +\alpha ^2)^{-\lambda }=\sum _{n=0}^{\infty }\mathcal {C}_{n}^{(\lambda )}(x)\alpha ^n. \end{aligned}$$

The case $\lambda =0$ is not considered here. $\{\mathcal {C}_{n}^{(\lambda )}\}_{n\in \mathbb {N}_0}$ are orthogonal with respect to the measure $(1-x^2)^{\lambda -1/2}\ \mathrm {d}x$ over $[-1,1]$, and by [4] [Eq. 8.930]:

1 $$\begin{aligned} \forall \lambda \in I_G: \mathcal {C}_{0}^{(\lambda )}(x)=1, \mathcal {C}_{1}^{(\lambda )}(x)=2\lambda x. \end{aligned}$$

In the tables [4] by Gradshteyn and Ryzhik five pages are devoted to various integrals of Gegenbauer polynomials, for they appear in many branches of mathematics and theoretical physics, but not all cases of interest are covered: in applications to meson physics in [5], integrals of the type

$$\begin{aligned} \int _{-1}^{1}\big [\mathcal {C}_{\ell }^{(\alpha )}(x)\big ]^2(1-x^2)^{\alpha -3/2-p}\ \mathrm {d}x, \end{aligned}$$

had to be evaluated for $\ell \in \mathbb {N}_0$, $p\in \mathbb {Z}$ and $\mathrm {Re}(\alpha -\frac{1}{2}-p)>0$, where the notation follows [6]. Later, Rashid obtained in [6] a general identity for

$$\begin{aligned} \int _{-1}^{1}\mathcal {C}_{\ell }^{(\alpha )}(x)\mathcal {C}_{k}^{(\beta )}(x)(1-x^2)^{\frac{\alpha +\beta -3}{2}-p}\ \mathrm {d}x, \end{aligned}$$

in terms of a hyper-geometric series$\ _4F_3$ evaluated at 1 for $\ell ,k\in \mathbb {N}_0$, $p\in \mathbb {Z}$ and $\mathrm {Re}\big ((\alpha +\beta -1)/2-p\big )>0$. Due to the close connection of zonal harmonics to Gegenbauer polynomials, similar integrals also appear in applications of determinantal point processes to energy estimates on spheres [2]. Yet the basic question of the $L^2$-norm of these polynomials has not been addressed in the literature, and we will fill this gap. The following notation will be used:

$$\begin{aligned} \Vert f\Vert _2^2:=\int _0^1 [f(x)]^2 \ \mathrm {d}x. \end{aligned}$$

We derive an asymptotic formula for $\Vert \mathcal {C}_{n}^{(\lambda )}\Vert ^2_2$ when $\lambda >0$ in Proposition 1. Indeed, one of the key ingredients in [1] was the asymptotic nature of $\Vert \mathcal {C}_{n}^{(2)}\Vert ^2_2$ in *n*, and the following lemma was proved in [1][Lemmas 6.1 and 6.2]:

### **Lemma 1**

*Let *$\psi$* denote the digamma function and* $\gamma$* the Euler–Mascheroni constant. Then the Gegenbauer polynomials satisfy for* $n\ge 2$:

$$\begin{aligned} \begin{aligned} \big \Vert \sqrt{1-x^2}\ \mathcal {C}_{n-2}^{(2)}\big \Vert _2^2&=\tfrac{1}{16}(2n^2-1)\big (\psi (n+\tfrac{1}{2})+\gamma +\log (4)\big ) -\tfrac{1}{8}n^2,\\ \big \Vert \mathcal {C}_{n-2}^{(2)}\big \Vert ^2_2&=\tfrac{1}{16}n^4+\tfrac{1}{64}(4n^2-1)\\&\quad \big (\psi (n+\tfrac{1}{2})+\gamma +\log (4)\big ) -\tfrac{5}{32}n^2. \end{aligned} \end{aligned}$$

*The following result of Corollary 5.2 from* [3]* will prove to be indispensable*.

### **Theorem 1**

(*Dette* [3])* The Gegenbauer polynomials satisfy for* $\lambda \in I_G$

2 $$\begin{aligned}&\left( \frac{n}{2 \lambda }\right) ^2\big [\mathcal {C}_{n}^{(\lambda )}(x)\big ]^2+ (1-x^2)\big [\mathcal {C}_{n-1}^{(\lambda +1)}(x)\big ]^2\nonumber \\&\quad = \sum _{k=0}^{n-1}\frac{\lambda +k}{\lambda }\big [\mathcal {C}_{k}^{(\lambda )}(x)\big ]^2. \end{aligned}$$

*Our main theorem is as follows, and we will use it to derive the asymptotic behavior of* $\Vert \mathcal {C}_{n}^{(\lambda )}\Vert ^2_2$.

### **Theorem 2**

*(Main Result) The Gegenbauer polynomials satisfy for* $\lambda \in I_G$:

$$\begin{aligned}&\big \Vert \mathcal {C}_{n-2}^{(\lambda +1)}\big \Vert _2^2=\frac{n^2-2\lambda n}{2^4\lambda ^3}\big [\mathcal {C}_{n}^{(\lambda )}(1)\big ]^2\\&\quad +\frac{ n(2n+1)}{2^3\lambda ^2}\big \Vert \mathcal {C}_{n}^{(\lambda )}\big \Vert _2^2-\sum _{k=0}^{n-1}\frac{\lambda +k}{2^2\lambda ^2}\big \Vert \mathcal {C}_{k}^{(\lambda )}\big \Vert _2^2. \end{aligned}$$

*From Theorem* 2 *we can deduce following more compact formulas.*

### **Corollary 1**

*The Gegenbauer polynomials satisfy for *$\lambda \in I_G$* and* $n>0$:

$$\begin{aligned}&\big \Vert \sqrt{1-x^2}\ \mathcal {C}_{n-1}^{(\lambda +1)}\big \Vert _2^2= \big [\mathcal {C}_{n}^{(\lambda )}(1)\big ]^2\ \frac{n+2\lambda }{n+1}\frac{1-2\lambda }{2^3\lambda ^2}\\&\quad +\frac{ (n+1)(2n+3)}{2^3\lambda ^2}\big \Vert \mathcal {C}_{n+1}^{(\lambda )}\big \Vert _2^2-\big \Vert \mathcal {C}_{n}^{(\lambda )}\big \Vert _2^2\ \frac{ n+2\lambda }{2^3\lambda ^2}. \end{aligned}$$

### **Corollary 2**

*The Gegenbauer polynomials satisfy for *$\lambda \in I_G$* and* $n>1$:

3 $$\begin{aligned} \begin{aligned}\big \Vert \mathcal {C}_{n-2}^{(\lambda +1)}\big \Vert _2^2=\frac{n^2-2\lambda n}{2^4\lambda ^3}\big [\mathcal {C}_{n}^{(\lambda )}(1)\big ]^2\\\quad +\frac{ 2n^2(4\lambda -1)+n(4\lambda +1)+2\lambda }{2^5\lambda ^3}\big \Vert \mathcal {C}_{n}^{(\lambda )}\big \Vert _2^2\\\quad -\big [\mathcal {C}_{n}^{(\lambda )}(1)\big ]^2\ \frac{n+2\lambda }{n+1}\frac{1-2\lambda }{2^5\lambda ^3}-\frac{ (n+1)(2n+3)}{2^5\lambda ^3}\big \Vert \mathcal {C}_{n+1}^{(\lambda )}\big \Vert _2^2. \end{aligned} \end{aligned}$$

### **Proposition 1**

*Let* $\mathcal {B}(x,y)$* denote the beta function. The following asymptotic formulas in n hold for* $\lambda \in (0,1)$* and* $\delta =\max \{4\lambda -1,2\lambda \}$:

$$\begin{aligned} \big \Vert \mathcal {C}_{n}^{(\lambda )}\big \Vert _2^2&<\ \mathcal {B}\big (1-\lambda ,\tfrac{1}{2}\big )\frac{2^{1-2\lambda }}{\Gamma (\lambda )^2}\frac{1}{n^{2-2\lambda }},\\ \big \Vert \mathcal {C}_{n}^{(\lambda +1)}\big \Vert _2^2&=\frac{n^{4\lambda }}{4\lambda \Gamma (2\lambda +1)^2}+O\big (n^{\delta }\big ),\\ \big \Vert \sqrt{1-x^2}\ \mathcal {C}_{n-1}^{(\lambda +1)}\big \Vert _2^2&<\frac{\ \mathcal {B}\big (1-\lambda ,\tfrac{1}{2}\big )}{\Gamma (\lambda +1)^2}\frac{n^{2\lambda }}{2^{1+2\lambda }}+O\big (n^{\delta -1}\big ). \end{aligned}$$

*The following asymptotic formulas hold for* $\lambda >1$* and* $\rho =\max \{4\lambda -3,2\lambda \}$:

$$\begin{aligned}&\big \Vert \mathcal {C}_{n-2}^{(\lambda +1)}\big \Vert _2^2=\frac{n^{4\lambda }}{4\lambda \Gamma (2\lambda +1)^2}+ \frac{\lambda -1}{\Gamma (2\lambda +1)^2}n^{4\lambda -1} +O(n^{4\lambda -2}),\\&\quad \big \Vert \sqrt{1-x^2}\ \mathcal {C}_{n-1}^{(\lambda +1)}\big \Vert _2^2=\frac{2\lambda -1}{4(\lambda -1)\Gamma (2\lambda +1)^2}n^{4\lambda -2}+O(n^{\rho }). \end{aligned}$$

*The identity* $2\cdot \Vert \mathcal {C}_{n}^{(1)}\Vert _2^2=\psi (n+\tfrac{3}{2})+\gamma +\log (4)$* is given *in [1][Eq. 14].

## Ingredients for the Proof of the Theorem

In this section we collect known results concerning Gegenbauer polynomials for later reference and the reader’s convenience, and we derive some technical lemmas in Subsect. 2.1 to prove Theorem 2. To avoid repetition, we will assume $\lambda \in I_G$ for the rest of the text if not stated otherwise. Note first that

4 $$\begin{aligned} \begin{array}{rll} \dfrac{\mathrm {d}}{\mathrm {d}x}\mathcal {C}_{n+1}^{(\lambda )}(x)&{}=2\lambda \ \mathcal {C}_{n}^{(\lambda +1)}(x)&{} {\text{[4, Eq. 8.935]}},\\ \mathcal {C}_{n}^{(\lambda )}(1)&{}=\frac{\Gamma (n+2\lambda )}{\Gamma (2\lambda )n!}= \frac{\prod _{j=1}^{n}(2\lambda +n-j)}{n!}&{} {\text{[4, Eq. 8.937]}};\\ \end{array} \end{aligned}$$

and $\mathcal {C}_{n}^{(\lambda )}(1)$ is the maximum on $[-1,1]$ for $\lambda >0$ by [7][Eq. 7.33.1]. Also, by (4):

5 $$\begin{aligned} (n+2)\ \mathcal {C}_{n+2}^{(\lambda )}(x)&=2\lambda \Big ( x\ \mathcal {C}_{n+1}^{(\lambda +1)}(x)-\mathcal {C}_{n}^{(\lambda +1)}(x)\Big )&{\text{[4, Eq. 8.933.2]}}, \end{aligned}$$

6 $$\begin{aligned} (n+\lambda )\ \mathcal {C}_{n}^{(\lambda )}(x)&=\lambda \Big (\mathcal {C}_{n}^{(\lambda +1)}(x)-\mathcal {C}_{n-2}^{(\lambda +1)}(x)\Big )&\,{\text{[4, Eq. 8.939.6]}}. \end{aligned}$$

### Identities for Gegenbauer polynomials

#### **Lemma 2**

*The Gegenbauer polynomials satisfy following identities*:

$$\begin{aligned} \begin{aligned}&\mathcal {C}_{n}^{(\lambda +1)}(x)+\mathcal {C}_{n-2}^{(\lambda +1)}(x)=2x\ \mathcal {C}_{n-1}^{(\lambda +1)}(x)+\mathcal {C}_{n}^{(\lambda )}(x),(\star )\\&\quad \int _0^1\big [\mathcal {C}_{n}^{(\lambda +1)}(x)\big ]^2-\big [\mathcal {C}_{n-2}^{(\lambda +1)}(x)\big ]^2\ \mathrm {d}x\\&\quad =\frac{n+\lambda }{2\lambda ^2}\Big (\big [\mathcal {C}_{n}^{(\lambda )}(1)\big ]^2+ (2\lambda -1)\big \Vert \mathcal {C}_{n}^{(\lambda )}\big \Vert _2^2\Big ).\\ \end{aligned} \end{aligned}$$

#### *Proof*

First we use (6) and apply (5) to the right-hand side below proving $(\star )$ while using the short-hand $\ell :=\lambda +1$:

$$\begin{aligned} \begin{aligned}&\mathcal {C}_{n}^{(\ell )}(x)+\mathcal {C}_{n-2}^{(\ell )}(x)=\frac{n+\lambda }{\lambda }\mathcal {C}_{n}^{(\lambda )}(x)\\&\quad +2x\ \mathcal {C}_{n-1}^{(\ell )}(x)-\Big (2x\ \mathcal {C}_{n-1}^{(\ell )}(x)- 2\mathcal {C}_{n-2}^{(\ell )}(x)\Big ). \end{aligned} \end{aligned}$$

Next we obtain by the binomial theorem with (6), $(\star )$ and (4)

$$\begin{aligned} \begin{aligned}&\big [\mathcal {C}_{n}^{(\lambda +1)}(x)\big ]^2-\big [\mathcal {C}_{n-2}^{(\lambda +1)}(x)\big ]^2\\&\quad =\frac{n+\lambda }{\lambda }\mathcal {C}_{n}^{(\lambda )}(x)\Big (2x\mathcal {C}_{n-1}^{(\lambda +1)}(x)+\mathcal {C}_{n}^{(\lambda )}(x)\Big )\\&\quad =\frac{n+\lambda }{\lambda }\Big (\frac{x}{2\lambda } \frac{\mathrm {d}}{\mathrm {d}x}\big [\mathcal {C}_{n}^{(\lambda )}(x)\big ]^2+\big [\mathcal {C}_{n}^{(\lambda )}(x)\big ]^2\Big ). \end{aligned} \end{aligned}$$

Integration by parts then finishes the argument. $\square$

#### **Lemma 3**

*The Gegenbauer polynomials satisfy the following identity:*

$$\begin{aligned} \begin{aligned}&\int _0^1x^2\big [\mathcal {C}_{n+1}^{(\lambda +1)}(x)\big ]^2+\big [\mathcal {C}_{n}^{(\lambda +1)}(x)\big ]^2\ \mathrm {d}x\\&\quad +\frac{1}{2\lambda }\int _0^1(1-x^2)\big [\mathcal {C}_{n+1}^{(\lambda +1)}(x)\big ]^2\ \mathrm {d}x\\&\quad =\frac{(n+2)^2}{8 \lambda ^3}\left[ \mathcal {C}_{n+2}^{(\lambda )}(1)\right] ^2+\frac{2\lambda -1}{2\lambda }\frac{(n+2)^2}{4 \lambda ^2}\big \Vert \mathcal {C}_{n+2}^{(\lambda )}\big \Vert _2^2. \end{aligned} \end{aligned}$$

#### *Proof*

Let $n=2m$. By Lemma 2 and a telescoping sum argument:

$$\begin{aligned} \begin{aligned}&\big \Vert \mathcal {C}_{n}^{(\lambda +1)}\big \Vert ^2_2-\big \Vert \mathcal {C}_{0}^{(\lambda +1)}\big \Vert ^2_2\\&\quad =\sum _{j=1}^m\frac{2j+\lambda }{2\lambda ^2}\Big (\big [\mathcal {C}_{2j}^{(\lambda )}(1)\big ]^2+ (2\lambda -1)\big \Vert \mathcal {C}_{2j}^{(\lambda )}\big \Vert _2^2\Big ),\\&\quad \big \Vert \mathcal {C}_{n+1}^{(\lambda +1)}\big \Vert ^2_2-\big \Vert \mathcal {C}_{1}^{(\lambda +1)}\big \Vert ^2_2\\&\quad =\sum _{j=1}^m\frac{2j+1+\lambda }{2\lambda ^2}\Big (\big [\mathcal {C}_{2j+1}^{(\lambda )}(1)\big ]^2 + (2\lambda -1)\big \Vert \mathcal {C}_{2j+1}^{(\lambda )}\big \Vert _2^2\Big ). \end{aligned} \end{aligned}$$

Using (1) and summing up, and an application of Dette’s result (2) yields:

$$\begin{aligned} \begin{aligned}&\int _0^1\big [\mathcal {C}_{n+1}^{(\lambda +1)}(x)\big ]^2+\big [\mathcal {C}_{n}^{(\lambda +1)}(x)\big ]^2\ \mathrm {d}x\\&\quad =\frac{4}{3}(\lambda +1)^2+1+\frac{1}{2\lambda }\sum _{j=2}^{n+1}\frac{j+\lambda }{\lambda }\big [\mathcal {C}_{j}^{(\lambda )}(1)\big ]^2\\&\quad\quad +\frac{2\lambda -1}{2\lambda }\sum _{j=2}^{n+1}\frac{j+\lambda }{\lambda }\big \Vert \mathcal {C}_{j}^{(\lambda )}\big \Vert _2^2\\&\quad =\frac{(n+2)^2}{8 \lambda ^3}\big [\mathcal {C}_{n+2}^{(\lambda )}(1)\big ]^2\\&\quad\quad +\frac{2\lambda -1}{2\lambda }\sum _{j=0}^{n+1}\frac{j+\lambda }{\lambda }\big \Vert \mathcal {C}_{j}^{(\lambda )}\big \Vert _2^2\\&\quad =\frac{(n+2)^2}{8 \lambda ^3}\big [\mathcal {C}_{n+2}^{(\lambda )}(1)\big ]^2\\&\quad\quad +\frac{2\lambda -1}{2\lambda }\Big (\frac{(n+2)^2}{4 \lambda ^2}\big \Vert \mathcal {C}_{n+2}^{(\lambda )}\big \Vert _2^2+\big \Vert \sqrt{1-x^2}\ \mathcal {C}_{n+1}^{(\lambda +1)}\big \Vert _2^2\Big ).\\ \end{aligned} \end{aligned}$$

The case $n+1=2m$ is analogous.$\square$

#### **Lemma 4**

*The Gegenbauer polynomials satisfy the following identity:*

$$\begin{aligned}&\int _0^1x^2\big [\mathcal {C}_{n+1}^{(\lambda +1)}(x)\big ]^2-\big [\mathcal {C}_{n}^{(\lambda +1)}(x)\big ]^2\ \mathrm {d}x\\&\quad = \frac{n+2}{4 \lambda ^2}\Big (\big [\mathcal {C}_{n+2}^{(\lambda )}(1)\big ]^2 -(n+3)\big \Vert \mathcal {C}_{n+2}^{(\lambda )}\big \Vert _2^2\Big ). \end{aligned}$$

#### *Proof*

Note first that by (5) and by quadratic completion

7 $$\begin{aligned} \begin{aligned}&2\frac{n+2}{2\lambda }\mathcal {C}_{n+2}^{(\lambda )}(x)\mathcal {C}_{n}^{(\lambda +1)}(x)\\&\quad =2x\mathcal {C}_{n+1}^{(\lambda +1)}(x)\mathcal {C}_{n}^{(\lambda +1)}(x)-2\big [\mathcal {C}_{n}^{(\lambda +1)}(x)\big ]^2\\&\quad=x^2\big [\mathcal {C}_{n+1}^{(\lambda +1)}(x)\big ]^2-\big [\mathcal {C}_{n}^{(\lambda +1)}(x)\big ]^2\\&\quad\quad -\Big (x\mathcal {C}_{n+1}^{(\lambda +1)}(x)-\mathcal {C}_{n}^{(\lambda +1)}(x) \Big )^2. \end{aligned} \end{aligned}$$

Hence by the binomial theorem and again by (5)

$$\begin{aligned} \begin{aligned}&2\int _0^1x^2\big [\mathcal {C}_{n+1}^{(\lambda +1)}(x)\big ]^2-\big [\mathcal {C}_{n}^{(\lambda +1)}(x)\big ]^2\ \mathrm {d}x\\&\quad =\frac{n+2}{\lambda }\int _0^1\Big (x\mathcal {C}_{n+1}^{(\lambda +1)}(x)+\mathcal {C}_{n}^{(\lambda +1)}(x)\Big )\mathcal {C}_{n+2}^{(\lambda )}(x)\ \mathrm {d}x\\&\quad =\frac{n+2}{\lambda }\int _0^1\frac{x}{4\lambda }\frac{\mathrm {d}}{\mathrm {d}x}\big [\mathcal {C}_{n+2}^{(\lambda )}(x)\big ]^2+\mathcal {C}_{n}^{(\lambda +1)}(x)\mathcal {C}_{n+2}^{(\lambda )}(x)\ \mathrm {d}x\\&\quad =\frac{n+2}{4\lambda ^2}\Big (\big [\mathcal {C}_{n+2}^{(\lambda )}(1)\big ]^2-\int _0^1\big [\mathcal {C}_{n+2}^{(\lambda )}(x)\big ]^2\ \mathrm {d}x\Big )\\&\quad\quad +2\frac{n+2}{2\lambda }\int _0^1\mathcal {C}_{n}^{(\lambda +1)}(x)\mathcal {C}_{n+2}^{(\lambda )}(x)\ \mathrm {d}x\\ \end{aligned} \end{aligned}$$

which proves the result when we substitute (7) and use (5) one last time.$\square$

## Proof of the main results

### *Proof of Theorem 2*

Subtract the left-hand sides of Lemma 3 and Lemma 4:

$$\begin{aligned} \begin{aligned}&2\big \Vert \mathcal {C}_{n}^{(\lambda +1)}\big \Vert ^2_2+\frac{1}{2\lambda } \big \Vert \sqrt{1-x^2}\ \mathcal {C}_{n+1}^{(\lambda +1)}\big \Vert _2^2\\&\quad =\Big (\frac{(n+2)^2}{8 \lambda ^3}- \frac{n+2}{4 \lambda ^2}\Big )\big [\mathcal {C}_{n+2}^{(\lambda )}(1)\big ]^2\\&\quad\quad +\Big (\frac{(n+2)^2}{4 \lambda ^2}+ \frac{(n+2)(n+3)}{4 \lambda ^2}\Big )\big \Vert \mathcal {C}_{n+2}^{(\lambda )}\big \Vert _2^2\\&\quad\quad -\frac{1}{2\lambda }\frac{(n+2)^2}{4 \lambda ^2}\big \Vert \mathcal {C}_{n+2}^{(\lambda )}\big \Vert _2^2; \end{aligned} \end{aligned}$$

an application of Dette’s formula (2) then gives the desired expression.$\square$

### *Proof of Corollary 1*

We use Lemma 4, add zero and obtain with Theorem 2

$$\begin{aligned}&\frac{n}{4 \lambda ^2}\Big (\big [\mathcal {C}_{n}^{(\lambda )}(1)\big ]^2 -(n+1)\big \Vert \mathcal {C}_{n}^{(\lambda )}\big \Vert _2^2\Big )\\&\quad\quad + \int _0^1(1-x^2)\big [\mathcal {C}_{n-1}^{(\lambda +1)}(x)\big ]^2\ \mathrm {d}x\\&\quad =\int _0^1\big [\mathcal {C}_{n-1}^{(\lambda +1)}(x)\big ]^2-\big [\mathcal {C}_{n-2}^{(\lambda +1)}(x)\big ]^2\ \mathrm {d}x\\&\quad =\frac{(n+1)^2-2\lambda (n+1)}{2^4\lambda ^3}\big [\mathcal {C}_{n+1}^{(\lambda )}(1)\big ]^2\\&\quad\quad +\frac{ (n+1)(2n+3)}{2^3\lambda ^2}\big \Vert \mathcal {C}_{n+1}^{(\lambda )}\big \Vert _2^2\\&\quad\quad -\sum _{k=0}^{n}\frac{\lambda +k}{2^2\lambda ^2}\big \Vert \mathcal {C}_{k}^{(\lambda )}\big \Vert _2^2\\&\quad\quad -\frac{n^2-2\lambda n}{2^4\lambda ^3}\big [\mathcal {C}_{n}^{(\lambda )}(1)\big ]^2-\frac{ n(2n+1)}{2^3\lambda ^2}\\&\quad \big \Vert \mathcal {C}_{n}^{(\lambda )}\big \Vert _2^2+\sum _{k=0}^{n-1}\frac{\lambda +k}{2^2\lambda ^2}\big \Vert \mathcal {C}_{k}^{(\lambda )}\big \Vert _2^2\\&\quad =\big [\mathcal {C}_{n}^{(\lambda )}(1)\big ]^2\\&\quad \Big ( \frac{(n+1)^2-2\lambda (n+1)}{2^4\lambda ^3}\frac{(n+2\lambda )^2}{(n+1)^2}-\frac{n^2-2\lambda n}{2^4\lambda ^3}\Big )\\&\quad\quad +\frac{ (n+1)(2n+3)}{2^3\lambda ^2}\big \Vert \mathcal {C}_{n+1}^{(\lambda )}\big \Vert _2^2-\big \Vert \mathcal {C}_{n}^{(\lambda )}\big \Vert _2^2\\&\quad \Big (\frac{ n(2n+1)}{2^3\lambda ^2}+\frac{\lambda +n}{2^2\lambda ^2}\Big )\\&\quad =\big [\mathcal {C}_{n}^{(\lambda )}(1)\big ]^2\ \frac{2n^2+3n+2\lambda -2\lambda n-4\lambda ^2}{2^3\lambda ^2(n+1)}\\&\quad\quad +\frac{ (n+1)(2n+3)}{2^3\lambda ^2}\big \Vert \mathcal {C}_{n+1}^{(\lambda )}\big \Vert _2^2-\big \Vert \mathcal {C}_{n}^{(\lambda )}\big \Vert _2^2\\&\quad \Big (\frac{ 2n^2+3n+2\lambda }{2^3\lambda ^2}\Big ). \end{aligned}$$

We re-order to obtain the result.$\square$

### *Proof of Corollary 2*

This follows directly from the proofs of Theorem 2 and Corollary 1.$\square$

### *Remark 1*

For our asymptotic analysis we will need the following identity, see [8]: For $|z|\rightarrow \infty$ and $\alpha ,\beta \ge 0$:

9 $$\begin{aligned}&\frac{\Gamma (z+\alpha )}{\Gamma (z+\beta )}=z^{\alpha -\beta }\nonumber \\&\quad \Big (1+\frac{(\alpha -\beta )(\alpha +\beta -1)}{2z}+O(|z|^{-2})\Big ), \end{aligned}$$

we obtain by (4) for $\lambda >0$:

10 $$\begin{aligned}&\Gamma (2\lambda )^2\cdot \big [\mathcal {C}_{n}^{(\lambda )}(1)\big ]^2=n^{4\lambda -2}\nonumber \\&\quad +2\lambda (2\lambda -1)n^{4\lambda -3} +O(n^{4\lambda -4}). \end{aligned}$$

### *Proof of Proposition 1*

We will write $\Vert \mathcal {C}_{n}^{(\lambda )}\Vert _2^2=\Theta (n^{\Phi (\lambda )})$ if there are some constants $c_1,c_2>0$ such that $c_1n^{\Phi (\lambda )}\le \Vert \mathcal {C}_{n}^{(\lambda )}\Vert _2^2 \le c_2n^{\Phi (\lambda )}$ for all *n* big enough. First we use (3) to show by induction that $\Phi (\lambda )$ exists for $\lambda >1$, and that $\big [\mathcal {C}_{n}^{(\lambda )}(1)\big ]^2=\Theta (n^{\Phi (\lambda )+2})$.

The case $\lambda =m\in \mathbb {N}_{>1}$: Lemma 1 gives the result for $\lambda =2$, and if it holds for *m*, then with (3) and abuse of notation we have:

$$\begin{aligned}&\big \Vert \mathcal {C}_{n-2}^{(m+1)}\big \Vert _2^2=n^2\ \Theta \big (n^{\Phi (m)+2}\big )+n^2\ \Theta \big (n^{\Phi (m)}\big )\\&\quad\quad +\Theta \big (n^{\Phi (m)+2}\big )+n^2\ \Theta \big (n^{\Phi (m)}\big ). \end{aligned}$$

This proves the claim as it shows that $\Vert \mathcal {C}_{n}^{(m+1)}\Vert _2^2=\Theta (n^{\Phi (m)+4})$, but by (4):

11 $$\begin{aligned} \mathcal {C}_{n}^{(\lambda +1)}(1)=\, & {} \frac{(2\lambda +n+1)(2\lambda +n)}{2\lambda (2\lambda +1)}\nonumber \mathcal {C}_{n}^{(\lambda )}(1)\\=\, & {} \Theta \big (n^2\mathcal {C}_{n}^{(\lambda )}(1)\big ), \end{aligned}$$

which, when squared and $\lambda =m$, is of order $\Phi (m)+6.$

The case $\lambda \in (m,m+1)$ for $m\in \mathbb {N}$: For $\lambda \in (0,1)$ and $\theta \in [0,\pi ]$:

$$\begin{aligned}\sin (\theta )^{\lambda }\big |\mathcal {C}_{n}^{(\lambda )}(\cos (\theta ))\big |< \frac{2^{1-\lambda }}{\Gamma (\lambda )} n^{\lambda -1}\,{\text {see [7, Eq. 7.33.5]}}.\end{aligned}$$

We square this inequality, multiply by $\sin (\theta )^{1-2\lambda }$ and integrate:

$$\begin{aligned}&\big \Vert \mathcal {C}_{n}^{(\lambda )}\big \Vert _2^2<\frac{2^{2-2\lambda }}{\Gamma (\lambda )^2} n^{2\lambda -2}\int _0^{\pi /2} \sin (\theta )^{1-2\lambda }\ \mathrm {d}\theta \\&\quad = \mathcal {B}\big (1-\lambda ,\tfrac{1}{2}\big )\frac{2^{1-2\lambda }}{\Gamma (\lambda )^2}n^{2\lambda -2}; \end{aligned}$$

where we used a change of variables $\theta =\arcsin (x)$ and $\mathcal {B}(x,y)$ is the beta function. This in combination with (10) and (3) gives for $\delta =\max \{4\lambda -1,2\lambda \}$:

12 $$\begin{aligned} \big \Vert \mathcal {C}_{n}^{(\lambda +1)}\big \Vert _2^2=\frac{n^{4\lambda }}{2^4\lambda ^3\Gamma (2\lambda )^2}+O\big (n^{\delta }\big ). \end{aligned}$$

Thus for $\lambda \in (0,1)$: $\Phi (\lambda +1)=4\lambda$, and $[\mathcal {C}_{n}^{(\lambda +1)}(1)]^2=\Theta \big (n^{4\lambda +2}\big )$ by (10), which finishes the case for the interval (1, 2), and we use induction with (3) and (11).

Hence, the two leading terms in the asymptotic form of $\Vert \mathcal {C}_{n}^{(\lambda +1)}\Vert _2^2$ are in the expansion of $\mathcal {C}_{n}^{(\lambda )}(1)$ when $\lambda >1$; using once more (10) and (3) yields

$$\begin{aligned}&\frac{n^2-2\lambda n}{2^4\lambda ^3}\big [\mathcal {C}_{n}^{(\lambda )}(1)\big ]^2= \frac{n^{4\lambda }}{4\lambda \Gamma (2\lambda +1)^2}\\&\quad + \frac{2\lambda (2\lambda -2)}{4\lambda \Gamma (2\lambda +1)^2}n^{4\lambda -1} +O(n^{4\lambda -2}). \end{aligned}$$

The asymptotic of the rest term follows by (3), Equation (12) and induction for non-integer $\lambda \in \mathbb {R}_{>2}$ or else Lemma 1 and induction when $\lambda \in \mathbb {N}_{> 2}$. These asymptotic formulas in combination with Corollary 1 and (10) will now finish the argument. We will only do the case $\lambda >1$, for the case $\lambda \in (0,1)$ is similar; let $\rho =\max \{4\lambda -3,2\lambda \}$:

$$\begin{aligned}&\big \Vert \sqrt{1-x^2}\ \mathcal {C}_{n-1}^{(\lambda +1)}\big \Vert _2^2= \frac{1-2\lambda }{2^3\lambda ^2}\big [\mathcal {C}_{n}^{(\lambda )}(1)\big ]^2\\&\quad\quad +\frac{ 2n^2}{2^3\lambda ^2}\Vert \mathcal {C}_{n+1}^{(\lambda )}\Vert _2^2+O(n^{\rho })\\&\quad = \frac{1-2\lambda }{2^3\lambda ^2}\frac{n^{4\lambda -2}}{\Gamma (2\lambda )^2}+\frac{ 2n^2}{2^3\lambda ^2} \frac{n^{4\lambda -4}}{4(\lambda -1)\Gamma (2\lambda -1)^2}+O(n^{\rho })\\&\quad = \frac{n^{4\lambda -2}}{2^3\lambda ^2\Gamma (2\lambda -1)^2}\Big (\frac{1-2\lambda }{(2\lambda -1)^2}+\frac{1}{2(\lambda -1)}\Big )+O(n^{\rho })\\&\quad = \frac{n^{4\lambda -2}}{2^3\lambda ^2\Gamma (2\lambda -1)^2}\frac{1}{(2\lambda -1)(\lambda -1)2}+O(n^{\rho }). \end{aligned}$$

$$\square$$

### *Remark 2*

One can use Lemma 1, Corollary 1 and Corollary 2 to find exact formulas for $\Vert \sqrt{1-x^2}\ \mathcal {C}_{n}^{(m)}\Vert _2^2$ and $\Vert \mathcal {C}_{n}^{(m)}\Vert _2^2$ where $m\in \mathbb {N}_{>1}$.
